# Functional assessment of human enhancer activities using whole-genome STARR-sequencing

**DOI:** 10.1186/s13059-017-1345-5

**Published:** 2017-11-20

**Authors:** Yuwen Liu, Shan Yu, Vineet K. Dhiman, Tonya Brunetti, Heather Eckart, Kevin P. White

**Affiliations:** 1 Institute for Genomics and Systems Biology, University of Chicago and Argonne National Laboratory, Chicago, IL 60637 USA; 2 Department of Human Genetics, University of Chicago, Chicago, IL 60637 USA; 3 Tempus Labs, 600 West Chicago Ave., Chicago, IL 60654 USA

**Keywords:** Enhancers, Regulatory elements, STARR-seq, Non-coding regions

## Abstract

**Background:**

Genome-wide quantification of enhancer activity in the human genome has proven to be a challenging problem. Recent efforts have led to the development of powerful tools for enhancer quantification. However, because of genome size and complexity, these tools have yet to be applied to the whole human genome.

**Results:**

In the current study, we use a human prostate cancer cell line, LNCaP as a model to perform whole human genome STARR-seq (WHG-STARR-seq) to reliably obtain an assessment of enhancer activity. This approach builds upon previously developed STARR-seq in the fly genome and CapSTARR-seq techniques in targeted human genomic regions. With an improved library preparation strategy, our approach greatly increases the library complexity per unit of starting material, which makes it feasible and cost-effective to explore the landscape of regulatory activity in the much larger human genome. In addition to our ability to identify active, accessible enhancers located in open chromatin regions, we can also detect sequences with the potential for enhancer activity that are located in inaccessible, closed chromatin regions. When treated with the histone deacetylase inhibitor, Trichostatin A, genes nearby this latter class of enhancers are up-regulated, demonstrating the potential for endogenous functionality of these regulatory elements.

**Conclusion:**

WHG-STARR-seq provides an improved approach to current pipelines for analysis of high complexity genomes to gain a better understanding of the intricacies of transcriptional regulation.

**Electronic supplementary material:**

The online version of this article (doi:10.1186/s13059-017-1345-5) contains supplementary material, which is available to authorized users.

## Background

Enhancers are DNA elements that control the temporal and spatial expression of genes and thus are central to biological processes such as development, differentiation, and homeostasis [1]. Enhancers maintain precise control of gene expression by serving as a loading platform to a variety of recruited transcription factors (TFs) and associated co-regulators that regulate productive transcription at core promoters [2, 3]. Recent studies have linked nucleotide variation in enhancer elements to a number of phenotypic changes, including human diseases [4]. As such, characterization of enhancers under various cellular contexts is of fundamental importance to understanding the genetic basis of development and disease pathogenesis.

Because of this fundamental importance of understanding enhancer function, the ENCODE Project and Epigenome Roadmap Consortiums have invested tremendous efforts into the generation of comprehensive predictive annotations of *cis-*regulatory elements located in non-coding regions of the genome [5, 6]. These datasets include, but are not limited to, analyses of TF-binding sites, histone modifications, and chromatin accessibility regions in various human tissues and cell types. These data are crucial for the identification of enhancers and for defining the epigenomic landscape in relation to enhancer functional impact on the regulatory programs that control development and disease. However, despite improvement on data-driven predictive algorithms in linking enhancers to their respective target genes, a proportionally small number of predictions have been functionally tested for enhancer activity. This lack of genome-wide prediction validation through enhancer functional activity assays can lead to many false-positive predictions [2, 7, 8]. Furthermore, these datasets can only identify putative enhancers based on TF and histone chromatin immunoprecipitation (ChIP-seq) data and therefore do not necessarily exhaustively define functional enhancers, leading to false negatives. With the discovery of thousands of nucleotide variants in these regulatory elements linked to potential phenotypic outcomes, these limitations of the current predictive approaches highlight the importance of obtaining genome-wide functional validations of enhancer activity.

Traditionally, enhancer activity has been quantified using reporter assays under the control of a minimal reporter to measure the activity of the cloned region of interest. While this method of quantification has proven to be reliable and accurate, until recently it was primarily used to serially investigate individual candidate sequences, making comprehensive assays of large genomes untenable. Recent advances have improved on this technique by developing enhancer reporter assays that combine massively parallel assays coupled with cell sorting techniques [9–11], molecular barcode sequencing [12–14], or by coupling the activity of a candidate enhancer to the abundance of its own self-transcribed sequence by placing candidate regions downstream of a core promoter and into the 3’UTR of a reporter gene [15]. The latter method, known as Self-Transcribing Active Regulatory Regions sequencing (STARR-seq), has allowed the assessment of enhancer activity on a genome-wide scale in model organism genomes and in selected regions of the human genome [2, 15–18].

While all of these massively parallel methods have profoundly improved on the traditional low-throughput enhancer reporter assay, they still provide only limited quantitative information and have not been scaled to the whole human genome, at which the ENCODE and Epigenomics Roadmap consortia have been performed. Scaling STARR-seq to a whole-genome level is a particularly promising prospect, as this technique allows active enhancers to transcribe themselves and become part of the resulting reporter signal and thus libraries can be constructed directly from human genomic DNA and do not require any complicated de novo synthesis steps with high error rate. However, there have been major limitations to the application of STARR-seq to the human genome, primarily due to the genome size and complexity of humans compared to *Drosophila*, where the technique was developed. This higher complexity requires more starting material and deeper sequencing depth to achieve a comparable power for detecting and quantifying enhancers in humans and hence demands method development to increase screening library complexity, scale up cell transfection, and increase efficiency of NGS library preparation. To date, the only STARR-seq applications that have been performed in human or other mammalian genomes are BAC-based [15] or capture-based approaches [19]. Unfortunately, these methods can only assay a small subset of predicted human enhancers. A method interrogating the entire human genome for enhancer activity has yet to be reliably developed.

In the current study, we demonstrate whole human genome STARR-seq (WHG-STARR-seq) on the human prostate cancer cell line, LNCaP, to generate the first whole-genome-scale enhancer mapping and activity quantification in human cell lines. We show that this unbiased enhancer screening allows the identification of active enhancers located not only in euchromatic regions, but also poised enhancers located in heterochromatic areas. Consequently, we find that the expression levels of nearby genes to these inaccessible enhancers are significantly lower than those near accessible enhancers, indicating that the inherent regulatory activity of enhancer elements may be epigenetically silenced by local chromatin structure. We test this hypothesis by treatment with the histone deacetylase inhibitor Trichostatin A (TSA), and we find the expression level of genes near previously heterochromatic enhancers to increase significantly. These data indicate that many enhancers have latent activities that can be readily uncovered by altering chromatin accessibility. This functional coordination between enhancer activity and chromatin accessibility, along with the existence of inaccessible enhancers, reinforce the idea that sequence-coded enhancer activity and chromatin context serve as two major functional layers that regulate transcription. This interplay is key for the understanding of non-coding variation as it contributes to human diseases and may have implications for how chromatin altering drugs might be combined with targeted therapies. WHG-STARR-seq scales the powerful STARR-seq methodology to the human genome and promises to help researchers reliably investigate regulatory element function in high complexity genomes elements.

## Results

### Generation of WHG-STARR-seq libraries

**Fig. 1:**
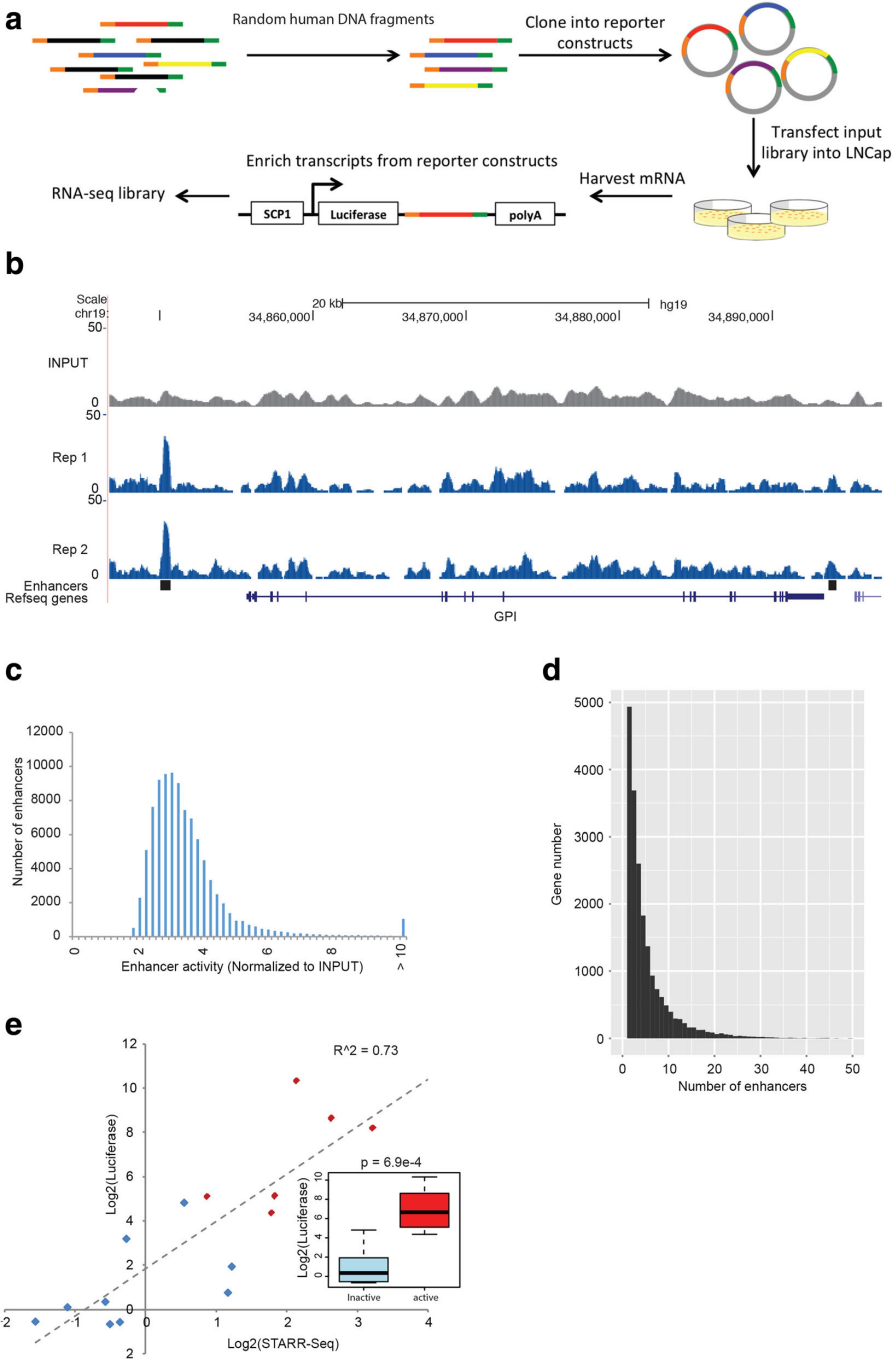
Experimental approach and validation of WHG-STARR-seq. **a** *Schematic representation* showing experimental setup and approach for WHG-STARR-seq. **b** Genomic *snapshot* displaying the *GPI* locus region as detected by WHG-STARR-seq. There is a strong enhancer region approximately 10 kb upstream of *GPI* transcriptional start site and another weaker enhancer region in the 3’UTR of *GPI*. Each *blue track* signifies normalized WHG-STARR-seq signal of each biological replicate. The *gray track* represents normalized WHG-STARR-seq signal of input library. **c** Distribution of WHG-STARR-seq enhancer activity of all detected enhancers. WHG-STARR-seq shows a wide dynamic range of enhancer signal normalized to INPUT (1.33–119.12, median = 3.08). **d** Distribution of number of detected enhancers associated per gene (enhancers are assigned to their nearest genes). **e** The enhancer activity of six active (*red*) and nine inactive (*blue*) enhancers were validated using traditional luciferase assays in biological triplicates. A strong correlation was observed between luciferase signal and WHG-STARR-seq enhancer activity

In order to identify DNA elements with regulatory potential across the entire human genome, we generated WHG-STARR-seq screening libraries by size selecting sheared DNA from random human genomic DNA (Fig. 1a). This approach allows us to interrogate the entire human genome, as contrasted to methods that rely on chromatin environment markers in combination with histone and TF ChIP-seq data to pre-select a small subset, typically on the order of thousands, of high-confident putative enhancers [15, 19–21]. We compared STARR-seq data using different minimal promoters, SCP1 and miniCMV, in our pilot study in another cell line MCF7. We found that under uniform experimental conditions, data obtained using the SCP1 promoter showed higher complexity than data obtained from miniCMV promoter (Additional file 1: Figure S1A, B). We also observed negligible differences in dynamic range of the assay in these two minimal promoter comparisons (Additional file 1: Figure S1B, C). Therefore, we decided to utilize the SCP1 promoter, with a similar configuration to that used in Arnold et al. [15] (Fig. 1a). Additionally, we developed a screening library generation protocol (see “Methods”) so that the screening libraries used in this study contained at least 158.6 million independent candidate fragments, with average fragment length size of 500 bp (Additional file 1: Figure S2). We were able to uniquely sequence 83.7% of the whole human genome. Of those regions, 74.3% had a sequencing depth of at least ten distinct fragments per nucleotide (Additional file 1: Figure S3). This unprecedented high complexity allows us to directly measure the regulatory activity of the majority of the human genome.

We used the human prostate cancer cell line, LNCaP, to demonstrate the utility of our screening library, due to the ease of handling and high transfection efficiency of LNCaP cells. We generated STARR-seq data from two biological replicates and pooled the replicates together for WHG-STARR-seq analyses, similar to the methods described in previous reports [15]. We identified 94,527 regions that were significantly enriched over the input library (MACS2, q-value < 0.05) (Fig. 1b). The signal of these enriched regions covers a large dynamic range where genes were associated with multiple enhancer regions and the median enhancer activity was 3.08 (Fig. 1c, d). Furthermore, the two biological replicates were highly correlated when comparing read counts and log-transformed read counts (*R* ^*2*^ = 0.93 and *R* ^*2*^ = 0.64, respectively; Additional file 1: Figure S5A, B), which is comparable to assay reproducibility of STARR-seq and CapStarr-seq assays. More than 90% of enhancers were either found in intergenic or intronic regions. Enhancers were also found in the 5’UTR and in the 3’UTR of nearby genes (Additional file 1: Figures S6, S7). A more stringent q-value cutoff (q-value < 0.01) only removed a couple hundred candidate peaks from the final analysis (Additional file 1: Figure S8). As such, for all further analyses, we decided to use a q-value cutoff of 0.05.

In order to validate the function of the active enhancers determined by WHG-STARR-seq, we selected 15 regions for all activity measurements and measured their activity using traditional *Renilla* luciferase reporter assays. These regions were previously tested for nuclear receptor binding in LNCaP cells in response to various hormone treatments independent of WHG-STARR-seq experiments. As seen in Fig. 1e, we observe a strong correlation (*R* ^*2*^ = 0.73) between enhancer activity and luciferase reporter signal, with most of the luciferase validated enhancers also showing strong WHG-STARR-seq enrichment (fold change > 2.0). Conversely, most regions that did not have positive WHG-STARR-seq enrichment did not test positive in the luciferase validation assay. These data demonstrate that the WHG-STARR-seq technique compares reasonably well to traditional luciferase reporter assays.

### Active enhancers correlate with both open and closed chromatin regions

**Fig. 2:**
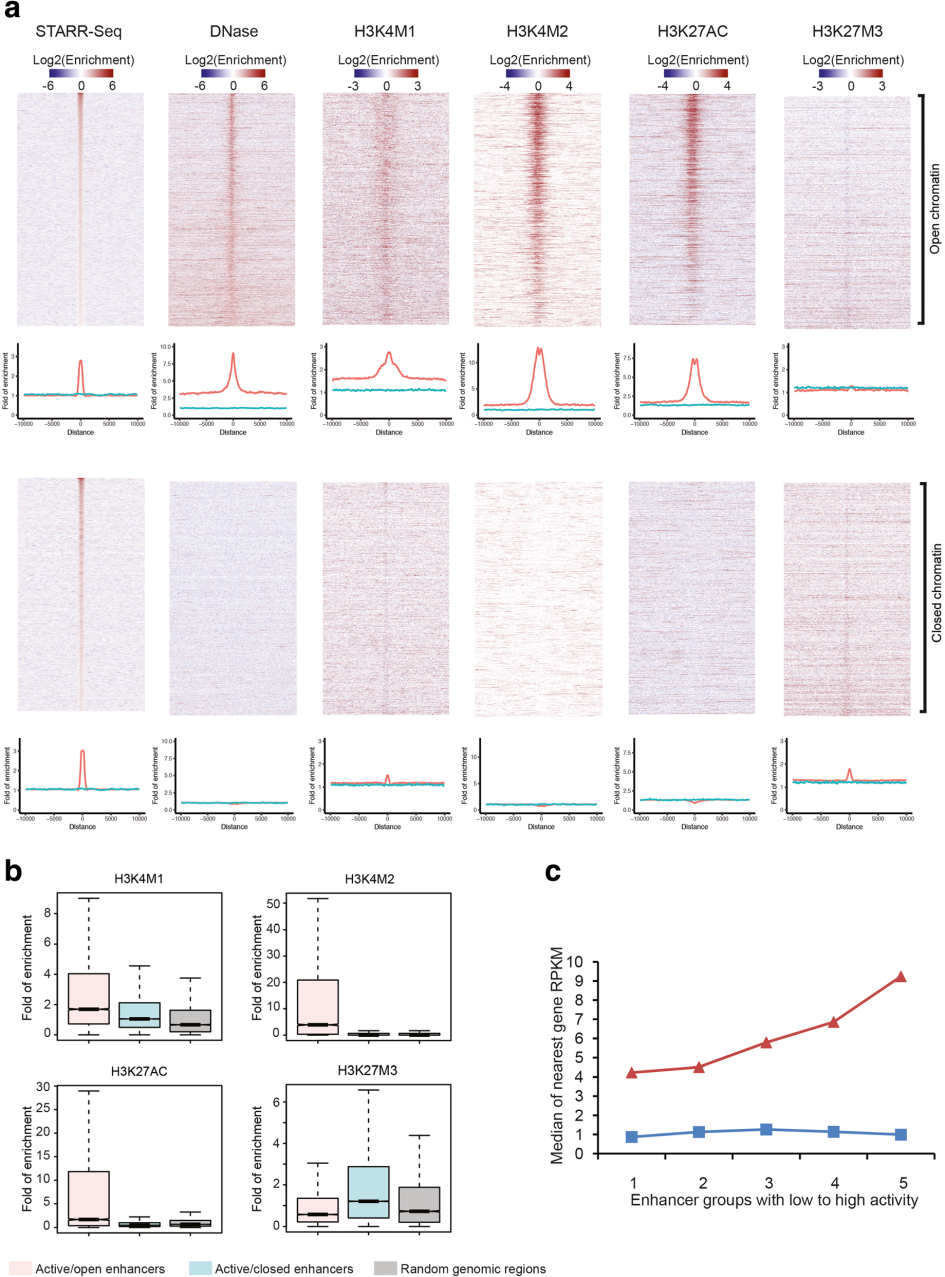
WHG-STARR-seq enhancers are associated with both open and closed chromatin environments. **a** *Heat maps* of various chromatin signal around WHG-STARR-seq enhancers. As generating high-resolution heat maps for all active open or active closed enhancers is too computationally intensive, we randomly sampled 5000 WHG-STARR-seq enhancers from open chromatin regions and from closed chromatin regions, respectively, and plotted signal of WHG-STARR-seq, DNase-seq, H3K4M1, H3K3M2, H3K27Ac, and H3K27M3 around these enhancers. We used all enhancers to make the *density plots* and *boxplots*. To make the density plots, we first extended the centers of enhancers to ± 10 kb, and then for each 100-bp tiling window in the extended regions, we calculated the fold enrichment of ChIP signal (normalized by library size) to INPUT signal (normalized by library size) across all windows. We plotted the average of the fold enrichment in the density plots (*red*: WHG-STARR-seq enhancers; *blue*: 10,000 random genomic regions). **b** *Boxplots* of fold enrichment of signal of various histone marks on WHG-STARR-seq enhancers (±200 bp around enhancer centers) and random genomic regions (±200 bp around random genomic region centers). **c** Association between gene expression level and nearby WHG-STARR-seq enhancers in open and closed chromatin regions. Enhancers were assigned to their nearest gene. All enhancers were binned into five separate groups according to the rank of enhancer signal (*red*: active/open enhancers; *blue*: active/closed enhancers)

Studies investigating transcriptional networks and regulatory elements tend to focus on euchromatic regions of the genome that allow access for TF and co-regulatory binding. However, because WHG-STARR-seq utilizes episomal reporter constructs, it allows the user to evaluate the potential of regulatory elements across the genome independent of their chromatin context. For example, of the six active WHG-STARR-seq enhancers validated by luciferase experiments, four correspond to open chromatin regions in LNCaP cells and two correspond to closed chromatin regions (Additional file 2: Table S1). This unbiased evaluation of enhancer activity potential provides the ability to investigate all regulatory elements and to uncover even deeper layers of transcriptional regulation that might become available under certain cellular or environmental conditions. To our surprise, only 12.7% (11,980) of the 94,527 active enhancers overlap with at least one of the 143,756 DNase I sites in the same cellular context, and thus were located in open chromatin regions (Additional file 1: Figure S9). The enhancers in open chromatin regions (active and open enhancers) are associated with active chromatin marks including H3K4me1, H3K4me2, and H3K27Ac (Fig. 2a, b, Additional file 1: Figure S10A). In addition, open chromatin regions with WHG-STARR-seq signal are observed to be more conserved than open chromatin regions without detectable WHG-STARR-seq signal (Additional file 1: Figure S11). The repressed chromatin mark H3K27me3 is depleted around active and open enhancers (Fig. 2a, b). Genes nearby these active enhancers in open chromatin display a positive correlation between enhancer activity and expression (Fig. 2c). Gene ontology (GO) term analysis of the top 2000 open active enhancers revealed genes enriched for house-keeping functions that include gene transcription, protein translation, and metabolic processes (Additional file 3: Table S2).

By contrast, the active regulatory elements identified by WHG-STARR-seq that are located in closed chromatin regions (active and closed enhancers) are not associated with DNase I signal, H3K4me2, or H3K27Ac marks (Fig. 2a, b, Additional file 1: Figure S10B), classifying them as having active potential, yet inaccessible in their native chromatin state. These regions do show weak but notable enrichment for H3K4me1 (Fig. 2a, b, Additional file 1: Figure S10B), consistent with previous studies that show H3K4me1 marks can be associated with enhancers regardless of their endogenous chromatin context [7, 22, 23]. These closed chromatin enhancers also show weak enrichment for H3K27me3 (Fig. 2a, b), indicating that some of these regulatory elements might be under the control of polycomb-mediated repression. For the closed chromatin enhancers, low presence of H3K4me1 may be indicative of poised enhancers, suggesting that these enhancers might be one stimuli away from active transcriptional regulation [24]. In further support of their inaccessibility, these closed chromatin regions do not show any correlation between enhancer activity and nearby gene expression level in LNCaP cells (Fig. 2c). Additionally, the conservation scores of closed, active enhancers that are opened in other ENCODE cells are higher than closed active enhancers that are closed in all ENCODE cell types (Additional file 1: Figure S12). Interestingly, the WHG-STARR-seq signal between accessible enhancers compared to inaccessible enhancers are similar to one another (Fig. 2a). In contrast to enhancers located in open chromatin regions, GO term analyses for nearby genes of enhancers located in closed chromatin revealed genes enriched for regulation various developmental processes (Additional file 3: Table S2). Motif enrichment analysis using HOMER [25] revealed that these enhancers are enriched for transcription factor binding sites such as FOXA1, NKX3.1, and STAT3 (Additional file 4: Table S3). Taken together, these results indicate that the vast majority of enhancers in the human genome are in epigenetically poised or silent states, but maintain regulatory potential.

### Closed chromatin enhancers drive nearby gene expression upon TSA treatment

**Fig. 3:**
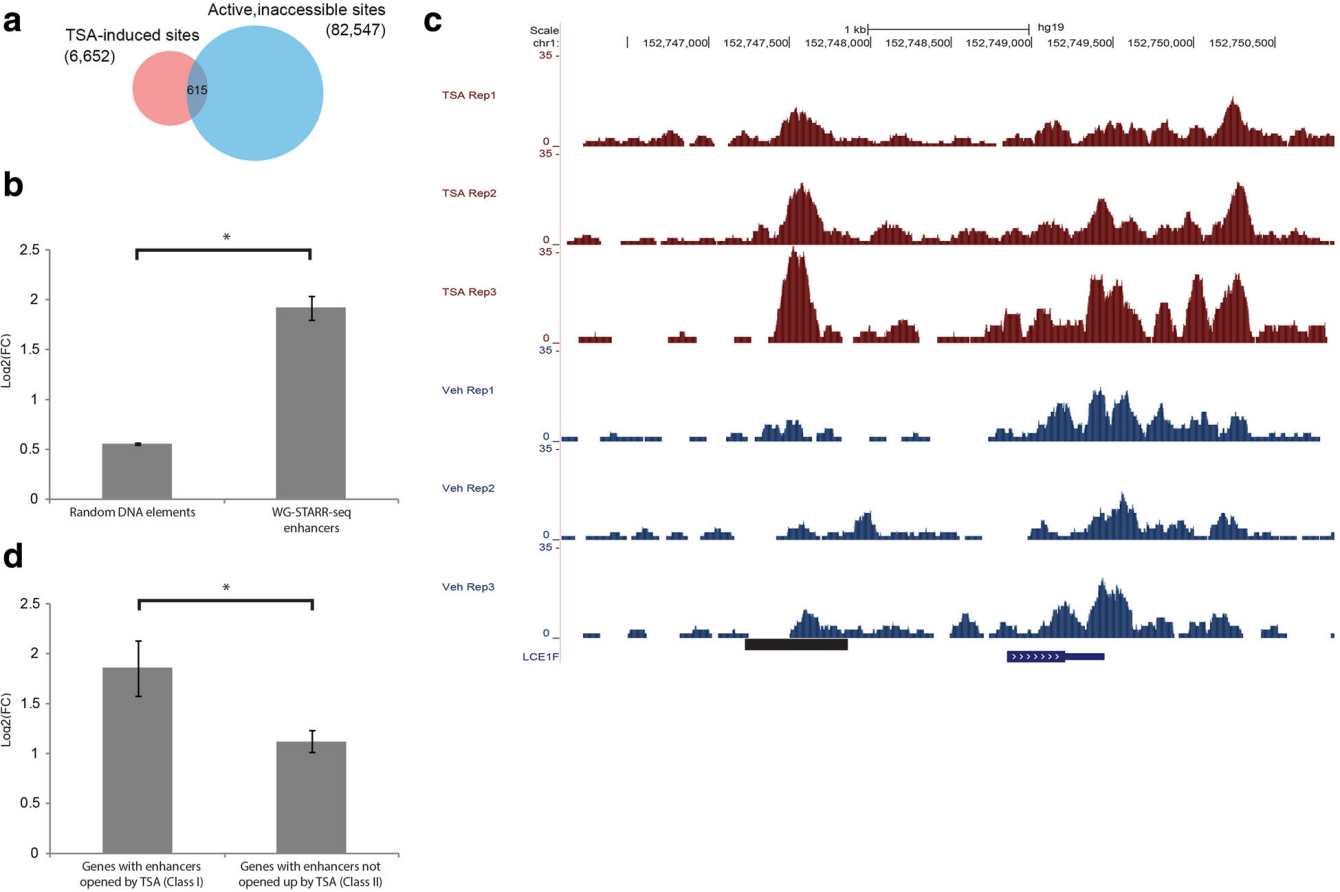
Inaccessible enhancers are functional upon treatment with TSA. **a** *Venn diagram* showing the overlap between active, inaccessible WHG-STARR-seq enhancers and TSA-induced sites. **b** Comparison of gene expression in response to TSA treatment of global genes and the 401 genes that have nearby WHG-STARR-seq enhancers which were inaccessible under vehicle treatment but became accessible under TSA treatment. Statistical significance was calculated using Wilcox Sum Rank Test (*p = 2.2e-16). **c** Genomic snapshot displaying ATAC-seq signal at *LCE1F* locus of LNCaP cells treated with TSA vs vehicle. **d** Comparison of nearby gene expression in response to TSA treatment of WHG-STARR-seq enhancers that were previously inaccessible, but opened up by TSA treatment (Class I) and regions not active in WHG-STARR-seq and opened up by TSA (Class II). Statistical significance was calculated by Wilcoxon sum rank test (*p = 1.6e-2)

To determine whether the active, inaccessible enhancers are able to regulate nearby gene expression upon a change in the endogenous chromatin environment, we treated LNCaP cells with TSA for 24 h. TSA is a histone deacetylase inhibitor (HDAC) that leads to an accumulation of acetylated histone, resulting in a relaxed chromatin environment. Following treatment with TSA, we performed assay for transposase accessible chromatin sequencing (ATAC-seq) and messenger RNA-sequencing (mRNA-seq) experiments to measure the genome-wide chromatic accessibility and gene expression levels, respectively. We identified 6652 sites (EdgeR [26], q-value < 0.05) with an observed increase in chromatin accessibility as compared to vehicle (we define these regions as TSA-induced sites hereafter). In inaccessible chromatin regions in the vehicle condition, there are 82,547 active, WHG-STARR-seq enhancers, among which 615 of these regions overlapped with the TSA-induced sites (Fig. 3a). By comparison, there are 2648 TSA-induced sites in closed chromatin regions without WHG-STARR-seq activity. Given that only ~ 3% of the closed chromatin regions have WHG-STARR-seq activity, there is a highly significant enrichment for active WHG-STARR-seq enhancers in regions with TSA-induced accessibility (*p* value = 7.4e-293). We assigned the 615 active, inaccessible enhancers with TSA-induced chromatin accessibility to their nearest genes and, as such, we found that 401 genes have at least one such enhancer. The expression level of these 401 genes are significantly higher when compared to all other genes across the genome (Fig. 3b). An example of this phenomenon can be seen with an enhancer region approximately 3-kb upstream of the *LCE1F* gene where, upon TSA treatment, there is a marked increase in chromatin accessibility in all three biological replicates (Fig. 3c).

To further statistically validate these experimental findings, we identified two different classes of genes. The first class (Class I) includes 59 genes that have nearby active, inaccessible enhancers (as defined by WHG-STARR-seq) opened up by TSA within 50 kb of their respective translational start sites. These genes also do not contain any nearby regulatory elements whose accessibility is decreased upon TSA treatment. The second class (Class II) of genes include 371 candidates that were selected with the same criteria, with the exception of being located nearby (within 50 kb) inaccessible DNA elements that were not identified by WHG-STARR-seq, but still opened up upon TSA treatment. Using a standard Wilcoxon statistical test, we find that the expression levels of genes in Class I are significantly higher than the expression of genes in Class II (*p* < 0.013) (Fig. 3d). Collectively, these results indicate that the closed chromatin enhancers identified by WHG-STARR-seq can become functional regulatory elements when their chromatin context assumes a more transcriptionally permissive environment.

### Interplay of chromatin context and enhancer strength in regulating nearby genes

**Fig. 4:**
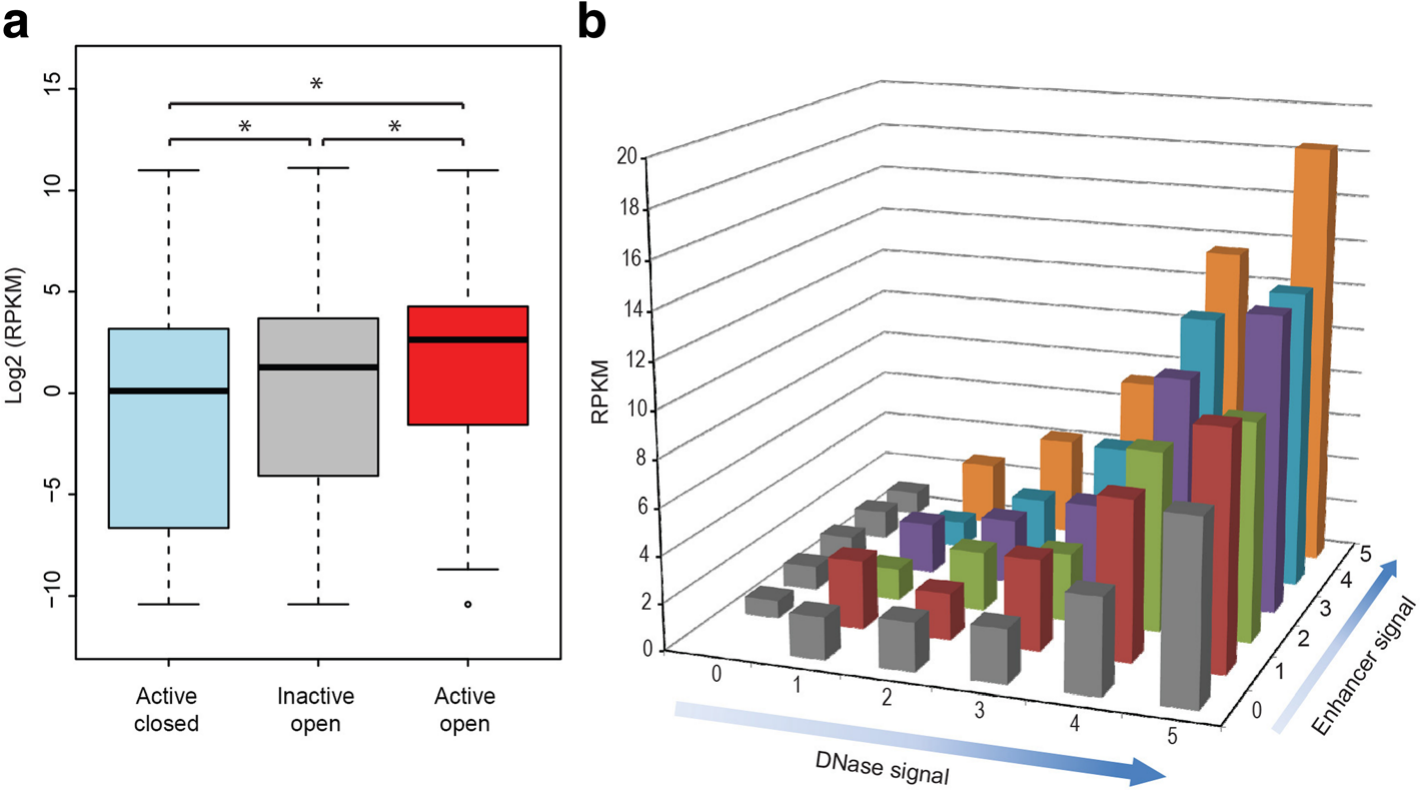
Gene expression levels are quantitatively associated with in chromatin accessibility and enhancer strength. **a** Comparison of expression levels of genes (denoted as RPKM) with different groups of enhancers nearby. Enhancers were assigned to their nearest gene (no distance cutoff). Statistical significance was calculated using Wilcox sum rank test (*p = 2.2e-16). **b** *3D plot* comparing expression levels of nearby genes in relation to both DNase I signal and enhancer activity. Both DNase I and enhancer signals are binned into six separate groups (0–5) according to the ranks of DNase I and enhancer signals, respectively

Based on the above results, we wanted to further investigate the relationship between chromatin context, enhancer strength, and nearby gene expression. While WHG-STARR-seq quantifies the inherent regulatory potential of enhancer elements, DNase-seq measures the accessibility of DNA elements to trans-acting factors such as TFs. As such, we wanted to integrate these two features to obtain a genome-wide assessment of their effects on gene expression levels. We first analyzed expression levels for genes nearby enhancers of varying activity and chromatin accessibility. Expression of genes near sequences located within closed chromatin, but that showed WHG-STARR-seq activity, was lower than the expression of genes near DNase I hypersensitivity sites that did not show WHG-STARR-seq enhancer activity (inactive and open sites) (Fig. 4a). This may be due to recalcitrance of some cis-regulatory elements in open chromatin regions to individual testing for functionality by reporter assay-based WHG-STARR-seq, or these elements may not correspond to enhancers but rather have other biological functions. As expected, we find the highest expression of genes nearby open chromatin enhancers identified by WHG-STARR-seq.

To obtain a more quantitative view of the relationship among WHG-STARR-seq enhancer activity, chromatin state, and gene expression, we categorized all active and accessible enhancers into 36 separate groups (ranked 0–5), based on their levels of DNase I signal and enhancer activity (Fig. 4b). Overall, we found that the average gene expression level of genes nearby these enhancers increases as one of the variables (DNase I signal or enhancer activity) stays fixed. Additionally, these two variables appear to act together such that higher enhancer signal and higher DNase I signal corresponded to genes with higher gene expression levels. Noticeably, enhancers with strong WHG-STARR-seq activity and weak DNase-seq signal were associated with lower gene expression levels than enhancers with weak WHG-STARR-seq activity and strong DNase-seq signal. This observation may reflect false negatives from the WHG-STARR-seq assay, high levels of basal promoter activity in some genes, or a combination of these factors. Overall these data indicate that the enhancers we validated with WHG-STARR-seq are indeed biologically relevant and are quantitatively predictive of gene expression output when coupled with chromatin state data.

## Discussion

In the current study, we improve on current enhancer profiling methods to develop WHG-STARR-seq, a technique that allows for the genome-wide, quantitative assessment of regulatory elements in large and high complexity genomes. This method allows for an unbiased analysis of the entire genome. Whereas previous methods were able to detect enhancers on the order of thousands in the human genome, WHG-STARR-seq is able to detect activity of tens of thousands of enhancers.

STARR-seq assays provide a complementary approach to orthogonal enhancer discovery methods. For example, a recent study by Inoue et al., observed substantial differences in chromosomal integrated libraries vs episomal libraries [27]. While integrated libraries do well in identifying endogenously active enhancers in a given cell or tissue type, our data in this study indicate that the detected enhancers from our episomal library are endogenously located in closed chromatin regions, accessible in other ENCODE cell lines and have biological relevance based on the sets of genes which reside nearby and the transcription factor binding sites enriched within them. Another complementary approach involves CRISPR screening, which has recently been shown to be effective for enhancer discovery [28, 29, 30] based on phenotypic readouts such as cell proliferation or survival to detect interesting regions for further interrogation. Such CRISPR mutational experiments demonstrate the necessity of specific genomic elements for enhancer activity, while STARR-seq demonstrates sufficiency as well as providing direct quantitative readout of enhancer activity.

Overall, we reproducibly detected 94,527 active enhancers in LNCaP cells. Surprisingly, the vast majority (87.3%) of active enhancers were found in closed chromatin regions. It has been suggested that the large size and complexity of the human genome, compared to organisms with substantially more compact genomes like *Drosophila*, corresponds to also to higher complexity of gene regulation [31]. Consistent with this idea, the fraction of enhancers mapped to closed chromatin regions in our data is about twofold of that in *Drosophila* STARR-seq data. Forty-one percent of these regions with WHG-STARR-seq activity, but endogenously within closed chromatin in LNCaP cells, reside in open chromatin regions of at least one other ENCODE cell type. Interestingly, the genes nearby these enhancers were enriched for organ developmental processes. These enrichment patterns provide biological relevance to this group of “poised” enhancers identified by WHG-STARR-seq. We hypothesize that these regions may likely play important roles in early organ development and afterwards become epigenetically silenced by histone modifications and/or chromatin remodeling. Thus, WHG-STARR-seq has the unique potential to identify hidden enhancers that, when exposed to trans-cellular environments, might act as pluripotent master regulators to reverse developmental processes. By comparison, we did find active WHG-STARR-Seq enhancers that are within closed chromatin for all ENCODE cell types. Near such enhancers, GO term analysis shows no biological process enrichment (data not shown). These enhancers also show low evolutionary conservation scores. Therefore, there is poor evidence supporting possible functional roles for active STARR-seq enhancers that are epigenetically silenced in all ENCODE cell types, but they might still affect biological processes when chromatin accessibility is artificially changed.

Furthermore, addition of a molecule (TSA) that causes the modification of a subset of chromatin marks in the genome revealed that many of these closed chromatin enhancers became endogenously functional and nearby genes increased in expression. This response to TSA clearly shows the interplay between chromatin structure and intrinsic enhancer activity in regulating transcriptional dynamics. Additionally, these observations illustrate limitations in methods and analyses that focus on using DNase I profiles and other chromatin mark datasets to predict and/or pre-select enhancer elements for functional characterization. Cellular environments in vivo can vary dramatically in response to a vast variety of variables including growth factors, cytokines, hormones, cell–cell contact, and physical or environmental challenges. Such variation in cellular environment may alter chromatin state, as demonstrated by the TSA experiments we performed to target histone modification enzymes. These results underscore that predictive methods that rely on chromatin state profiling for predicting enhancers genome-wide in the ENCODE and related projects are likely limited by the conditions under which cells are cultured.

## Conclusions

While STARR-seq has efficiently covered enhancer activity in flies [15] and CapStarr-seq improved on existing methods for the detection of a subset of active enhancers in the mouse genome [19], we have been able to successfully modify and extend this enhancer profiling approach to the entire human genome. WHG-STARR-seq promises to provide an unprecedented amount genome coverage for assessment of regulatory elements and transcriptional programs in many diverse human cellular systems, and in response to multiple cellular conditions.

## Methods

### Cell culture

LNCaP cells were maintained in RMPI 1640 media (ThermoFisher Scientific), 10% FBS (Gemini) and 1.0% penicillin/streptomycin (ThermoFisher Scientific).

### Generation of input plasmid library

Generation of plasmid libraries was based on the method described by Arnold et al. [15]. Briefly, human genomic DNA (Promega) was sheared by sonication (Covaris S2) and size-selected on 1% agarose gel (350–650 bp). Size-selected DNA fragments were ligated to adaptors (sense: 5’-ACACTCTTTCCCTACACGACGCTCTTCCGATCT-3’; antisense: 5’-GATCGGAAGAGCACACGTCT-3’) and polymerase chain reaction (PCR) amplified using Q5 High-Fidelity DNA polymerase (NEB) to add homology arms for cloning (forward primer: 5’-GTAATAATTCTAGAGTCGGGGCGGGAATGATACGGCGACCACCGAGATCTACACTCTTTCCCTACACGACGCTCTTCCGATCT-3’; reverse primer: 5’- TATCATGTCTGCTCGAAGCGGCATAGTGACTGGAGTTCAGACGTGTGCTCTTCCGATCT-3’; PCR program: 98 °C for 30 s; 12 cycles of 98 °C for 10 s, 65 °C for 30 s, and 72 °C for 30 s). The vector backbone was modified from pGL4.23[*luc2/minP*] (Promega). Briefly, minP promoter was replaced with SCP1 promoter and a CmR-ccdB cassette was cloned between luc2 ORF and SV40 poly (A). The PCR product (homology arms added to DNA fragments) was introduced into the vector backbone (linearized by SphI and NdeI restriction enzymes) using Gibson Assembly Master Mix (NEB), followed by transformations into MegaX DH10B™ T1^R^ electrocompetent cells (ThermoFisher Scientific). A total of 32 Gibson Assembly and transformations were performed. All transformed *E. coli* cells were pooled to grow in 4 L LB_AMP_ medium and harvested when Optical Density (OD) reached 1.0. The input plasmid library was extracted using Plasmid Mega Kit (Qiagen) and drop dialyzed before transfection.

### Transfection of input plasmid library into LNCaP cells

Input plasmid library was electroporated into LNCaP cells using the Nucleofector™ platform (Lonza) according to manufacturer’s protocol (1 μg DNA/1 million cells). A total of 300 million cells were electroporated in each replicate and two biological replicates were generated. After transfection, cells were plated in phenol red free RPMI 1640 medium (ThermoFisher Scientific) supplemented with 10% charcoal-stripped FBS (Gemini) and 1.0% penicillin/streptomycin (ThermoFisher Scientific) for 72 h until harvest.

### WHG-STARR-seq library preparation

Total RNA was extracted from transfected LNCaP cells using PerfectPure RNA Cultured Cell Kit (5PRIME), and followed by poly (A) tail mRNA isolation using Dynabeads® Oligo (dT) kit (ThermoFisher Scientific). mRNA was treated with TURBO™ DNase (ThermoFisher Scientific) and purified by Agencourt RNAClean XP beads (Beckman Coulter). Target-specific first strand complementary DNA (cDNA) synthesis was performed with SuperScript III (ThermoFisher Scientific; 1.5 μg mRNA/reaction) to specifically reverse transcribe WHG-STARR-seq mRNAs (RT primer: 5’- CAAACTCATCAATGTATCTTATCATG-3’). All mRNA from the 150 million transfected cells was used in reverse transcription. After reverse transcription, reactions were treated with RNase A + H and pooled. In the final PCR amplification, every 5 μL cDNA reaction was used as a template in every 50 μL PCR reaction using Q5 High-Fidelity DNA polymerase (NEB) using the following program: 98 °C for 30s; 20 cycles of 98 °C for 10 secs, 65 °C for 30 secs and 72 °C for 30 secs. Illumina HiSeq platform compatible primers were used (forward: 5’- AATGATACGGCGACCACCGAGATCTACACTCTTTCCCTACACGACGCTCTTCCGATCT-3’; reverse: 5’-CAAGCAGAAGACGGCATACGAGAT-index-GTGACTGGAGTTCAGACGTG-3’). For each replicate, we set up separate final PCR reactions using 16 different indexing primers. A total of 20 duplicated PCR reactions were performed for each indexing PCR. PCR products were cleaned up by AmPure XP beads (Beckman Coulter; beads/reaction ratio = 0.8) and submitted fro Illumina sequencing. Input library for sequencing was generated using similar PCR setup. Products from 12 duplicated PCR reactions were pooled (10 ng plasmid/reaction, ten cycles of PCR).

STARR-seq consists of two parts of data with different types of readouts. The input library is generated by directly amplifying inserts from the plasmid DNA used for cell transfection, served as reference of the original representation of insert fragments in starting plasmid pool. The output library measures the abundance of self-transcribed mRNA from insert fragments of the transfected plasmid pool.

### Luciferase reporter assay

The vector backbone for the reporter assay used the input plasmid library backbone without a CmR-ccdB cassette. Candidate regions were PCR amplified from human genomic DNA (Promega) and cloned into the vector through Gibson Assembly reactions (vector linearized by KpnI and NheI restriction enzymes). Reporter plasmids were co-transfected with luciferase control vector pRL-CMV (Promega) into cells with Lipofectamine® 2000 (ThermoFisher Scientific) following the manufacturer’s protocol (1.0 × 10^5^ cells/replicate, three replicates/testing region). After transfection, cells were seeded in 24-well plates in phenol red free RPMI 1640 medium (ThermoFisher Scientific) supplemented with 10% charcoal-stripped FBS (Gemini) and 1% penicillin/streptomycin (ThermoFisher Scientific). Dual luciferase assays (Promega) were performed at 72 h post transfection following manufacturer’s protocol.

### Trichostatin A treatment

Seeded in six-well plates were 1.0 × 10^5^ cells per well in phenol red free RPMI 1640 medium (ThermoFisher Scientific) supplemented with 10% charcoal-stripped FBS (Gemini) and 1% penicillin/streptomycin (ThermoFisher Scientific) for 72 h before treatment. Cells were treated with either 100 ng/mL TSA (Sigma Aldrich) or DMSO vehicle for 24 h before harvest to achieve three biological replicates.

### ATAC-seq and RNA-seq library preparation

ATAC-seq was performed as previously described [32]. Nuclei from 50,000 TSA/DMSO treated cells were freshly extracted and incubated with Tn5 transposase (Illumina). Transposed DNA was extracted from nuclei and PCR amplified using NEBNext High-Fidelity DNA polymerase (NEB) and customized Nextera PCR primers.

Total RNA was extracted from TSA/DMSO treated cells using PerfectPure RNA Cultured Cell Kit (5PRIME). RNA-seq library preparation was conducted using TruSeq Stranded mRNA Library Prep Kit (Illumina) according to manufacturer’s protocol (1 μg total RNA was used as initial material).

### Enhancer peak calling

Paired-end sequencing reads (2 × 100 bp) from Illumina Hiseq2000 were aligned to human genome hg19 by bowtie. Reads that are mapped to more than one location to the genome were filtered out. To exclude potential PCR amplification bias, fragments (inferred from paired-end reads) that have the same start and end positions were collapsed into distinct fragments by Picard (http://broadinstitute.github.io/picard/). Uniquely mapped distinct fragments from the 16 different indexing libraries were pooled and all included for downstream analysis, since duplicated fragments coming from different indexing libraries are considered as biological duplicates. To identify enhancers, we combined data from two biological replicates and called enhancer peaks by MACS2 (default setting, q-value < 0.05) [33]. An enhancer peak is called when there is significant enrichment of fragments from one region in output library than the representation of that region in input library based on Poisson distribution using MACS2 (false discovery rate < 0.05). The genome coverage of the plasmid library was used as input when calculating the enrichment of STARR-seq reads. We took the enrichment score reported by MACS2 as enhancer activity. Enhancer activity for a given peak was calculated as (number of distinct fragments in peak region in output library scaled by library size)/(number of distinct fragments in peak region in input library scaled by library size).

### Addressing duplicate fragments

Since it is difficult to dissect biological duplicates from PCR/sequencing duplicates in the sequencing reads and using total reads may inappropriately inflate statistical power in peak detection leading to false positives, we decided to use distinct fragments for peak calling and downstream analyses, similar to previous studies [15, 19]. However, it is likely that some highly active enhancers are underestimated. To increase peak calling sensitivity without introducing PCR/sequencing artifacts, we modified the library preparation protocol and data processing pipeline of the original STARR-seq paper. For each replicate of output library, we set up separate final PCR reactions using 16 different indexing primers and submitted for Illumina sequencing. We aligned reads to human genome and extracted uniquely mapped distinct fragments from the sequencing data of the 16 different indexing libraries, separately. Then we pooled all distinct mapped fragments from the 16 libraries and used all pooled fragments for downstream peak calling and analyses. Duplicated fragments coming from different indexing libraries could be justified as biological duplicates, which should be all included for analysis. Though an optimal solution is needed to barcode each single cDNA molecule in order to get unbiased estimation, we have increased the power from the original paper.

### Comparing enhancer activity with histone marks, DNase I signal, and gene expression data

To compare whole-genome STARR-seq enhancer signals with other epigenomics features, we gathered raw sequencing data for H3K4Me2 (GSE43720), H3K27Me3 (GSE62497), H3K27Ac (GSE51621), DNase I (GSE32970), and chromatin input (GSE62497, GSE51621) in LNCaP cells under vehicle-treated conditions. We combined the datasets for each epigenomics feature together and mapped the reads to human genome hg19 by Bowtie [34], filtered out reads that have multiple alignment, and collapsed reads with the same genomic start and end sites. We used DNase I peaks called through the ENCODE pipeline (GSE32970) to characterize chromatin accessibility in LNCaP cells under vehicle condition. Active STARR-Seq enhancers that overlap with open chromatin peaks are defined as open active enhancers and those that do not overlap are defined as closed active enhancers. In order to study the association of epigenomics marks with enhancer activity, we aligned enhancers within each of these two classes based on their centers and extended 10 kb to both directions. Reads from each feature were normalized to the same 50-M sequencing depth. We then calculated the mean enrichment of epigenomics reads over input reads (reads from each feature were first normalized to the same 50-M sequencing depth) in each 100-bp non-overlapping window spanning 20 kb around the centers of enhancers. Enhancers were assigned to their nearest genes by Homer [25] when comparing enhancer activity with gene expression.

### Processing of mRNA-seq data

Single-end reads of 50 bp from Illumina Hiseq2000 were mapped to the human genome hg19 by Tophat [35]. Reads were intersected with gene annotations from Refseq and RPKM values were calculated as estimates of gene expression levels. To study the transcriptional responses after TSA treatment, we first counted reads for each gene under each condition and then used EdgeR [26] (GLM tagwise dispersion used, q-value < 0.05) to identify genes that were differentially regulated comparing TSA to vehicle. All mRNA-seq experiments were performed with three biological replicates.

### Processing of ATAC-seq data

Paired-end reads (2 × 50 bp) were sequenced by Illumina Hiseq2000. Because of the existence of adaptor sequences in library molecules generated by ATAC-seq insertion events within 50 bp, we used Bowtie and Bowtie2 [36] for each library to do a two-round alignment to the hg19 human genome. In the first round, Bowtie was used and only uniquely mappable reads were kept and unmappable reads were gathered as the input for the second round. In the second round, after removing adaptor sequences by Cutadapt [37] (CTGTCTCTTATACACATCTCCGAGCCCACGAGACNNNNNNNNATCTCGTA in Read 1, CTGTCTCTTATACACATCTGACGCTGCCGACGAGTGTAGATCTCGGTGGT in Read 2), Bowtie2 was used for alignment and uniquely mappable reads were retained. We used Bowtie2 in the second round because Bowtie was not able to map pairs of reads that are complementary to each other, which arose from two adjacent ATAC-seq insertion events within 50 bp. Subsequently, we removed potential PCR duplicates by running Picard. We then adjusted the read start sites to represent the center of transposon binding event. All uniquely mappable reads aligning to the plus strand were offset by + 4 bp and those aligning to the minus strand were offset by − 5 bp.

To identify TSA-induced chromatin accessibility change, we first used Fseq [38] at default parameters (http://fureylab.web.unc.edu/software/fseq/) to call peaks in both vehicle condition and TSA treatment. We then merged peaks across conditions and counted the ATAC-seq reads number for each peak in each condition. Genomic regions showing differential accessibility after TSA treatment were called by EdgeR (we treated each region as if it was a gene, GLM tagwise dispersion used, q-value < 0.05). Three biological replicates were collected for each condition. We then overlapped closed active enhancers to TSA-induced sites to find closed, active enhancers that are opened up by TSA treatment.

## Additional files

Additional file 1: Supplemental figures. (PDF 10391 kb)

Additional file 2: Table S1. Genomic coordinates and chromatin environment of the 15 regions validated by luciferase assay. (XLSX 43 kb)

Additional file 3: Table S2. GO term analysis of the top 2000 active open enhancers and the top 2000 active closed enhancers. (XLSX 37 kb)

Additional file 4: Table S3. Motif enrichment analysis of WHG-STARR-seq enhancers. (XLSX 24 kb)
