# Supplementary material for: Functional assessment of human enhancer activities using whole-genome STARR-sequencing
Source: Genome Biol. 2017 Nov 20;18:219. doi: 10.1186/s13059-017-1345-5 (PMC5694901; doi:10.1186/s13059-017-1345-5)
Supplement: Supplementary file 1 — Supplemental figures. (PDF 10391 kb) [file 13059_2017_1345_MOESM1_ESM.pdf]

A

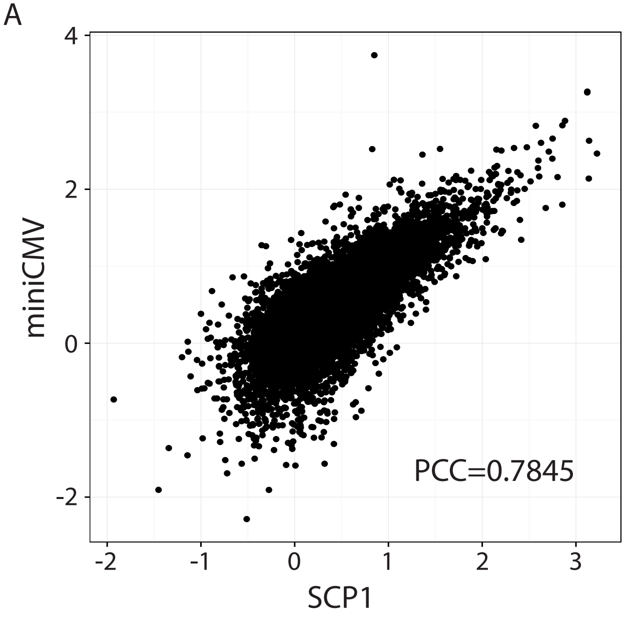

B

Promoter	Mean No. of distinct fragments	Median No. of distinct fragments	Regions with >= 10x coverage
SCP1	1.24×10^6	1.3×10^6	88.4%
miniCMV	3.3×10^5	3.2×10^5	48.3%

C

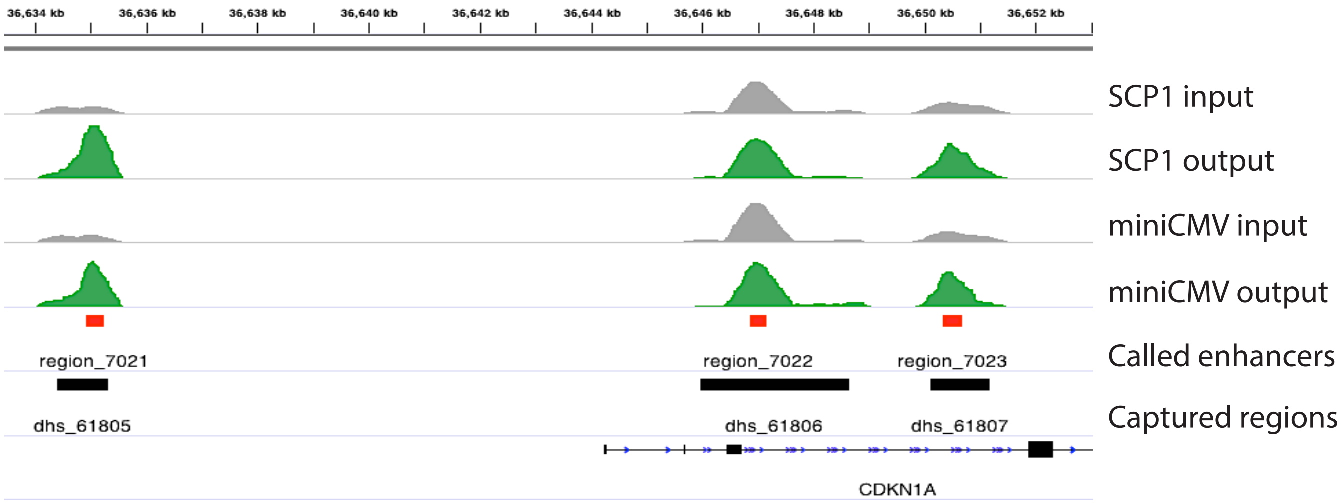

Figure S1: Comparison of SCP1 and miniCMV promoters in CapStarr-seq data in MCF7 cells. A) Correlation of enrichment signal between SCP1 and miniCMV data. **B)** Complexity of CapStarr-seq output libraries using different promoters. **C)** Snapshot of side-by-side comparison of SCP1 and miniCMV CapStarr-seq signal.

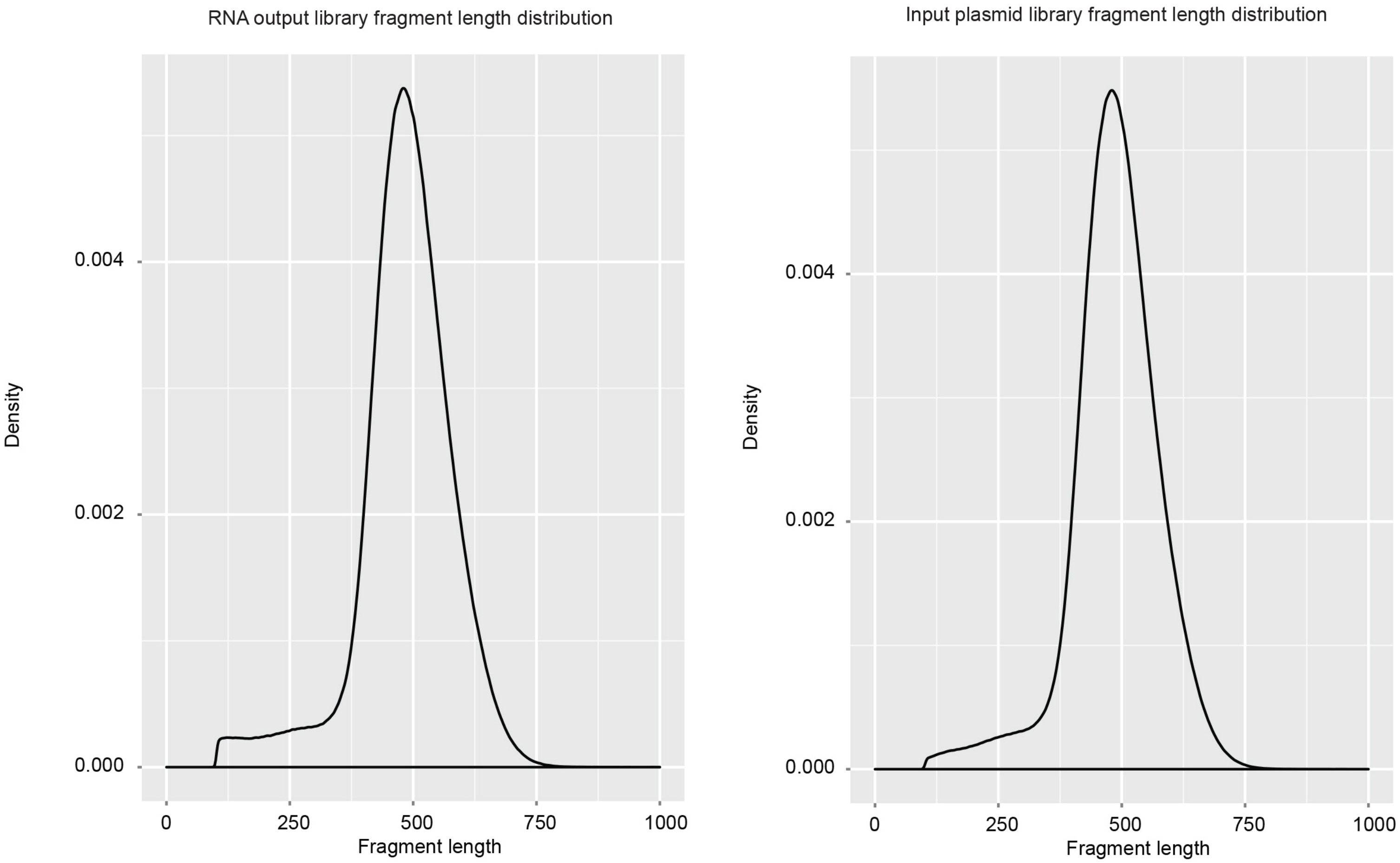

Figure S2: Distribution of fragment lengths of WHG-STARR-seq output and input libraries

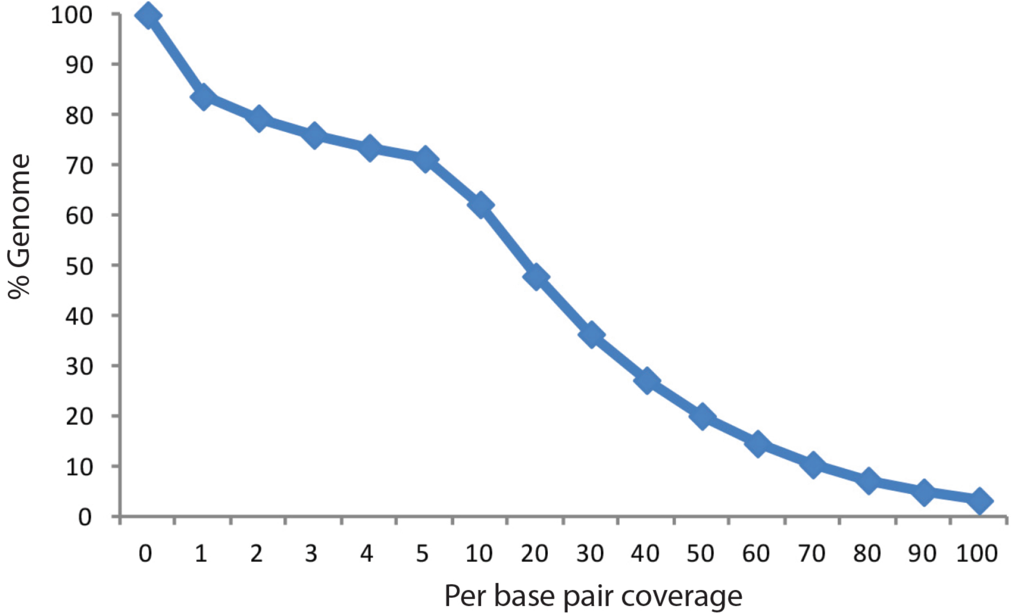

Figure S3: Cumulative plot of input screening library per base pair coverage in the genome

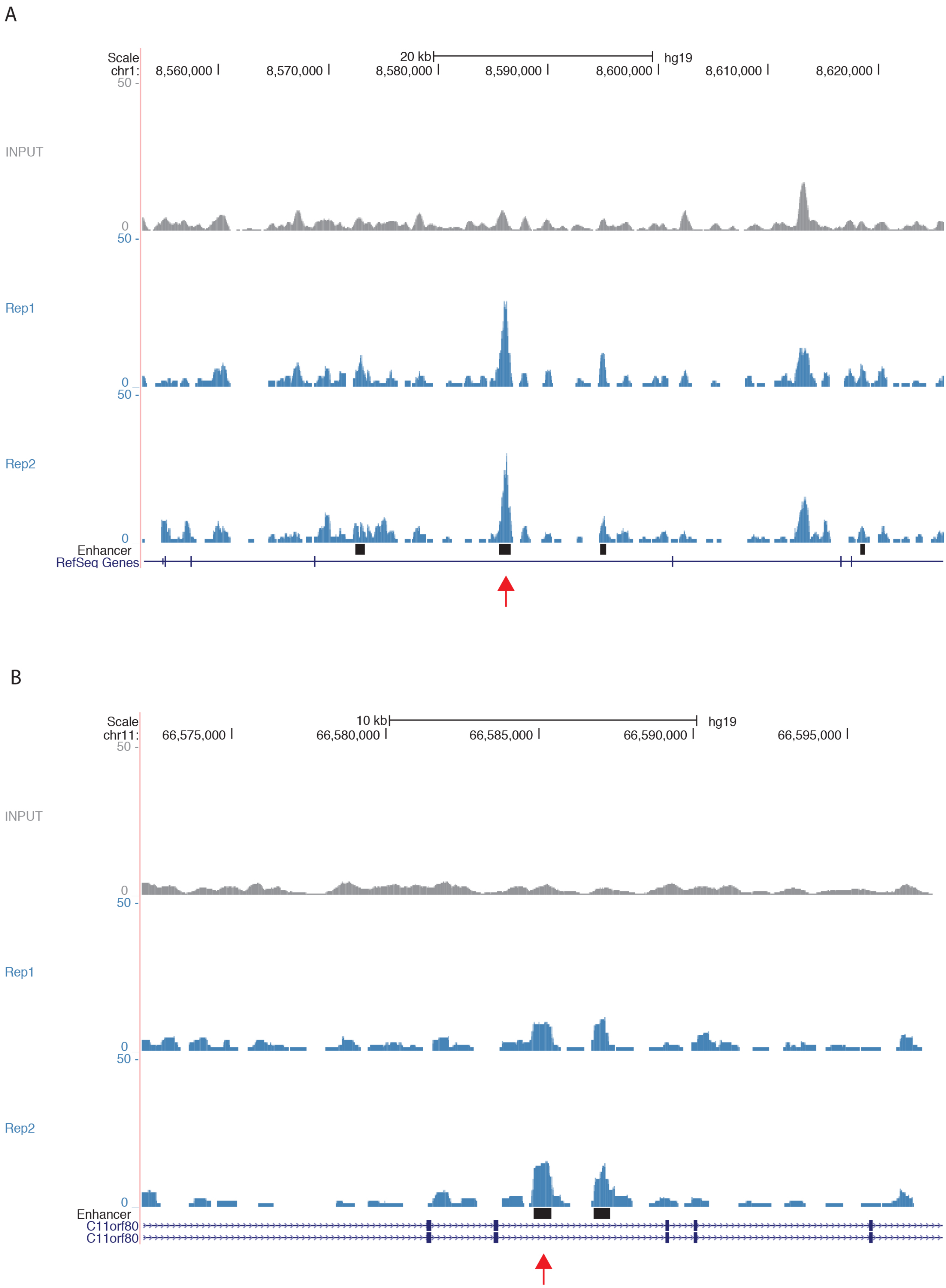
A
Scale
chr1:
20 kb
hg19
8,560,000
8,570,000
8,580,000
8,590,000
8,600,000
8,610,000
8,620,000
50
0
INPUT
Rep1
Rep2
Enhancer
RefSeq Genes
B
Scale
chr11:
10 kb
hg19
66,575,000
66,580,000
66,585,000
66,590,000
66,595,000
INPUT
Rep1
Rep2
Enhancer
C11orf80
C11orf80

C

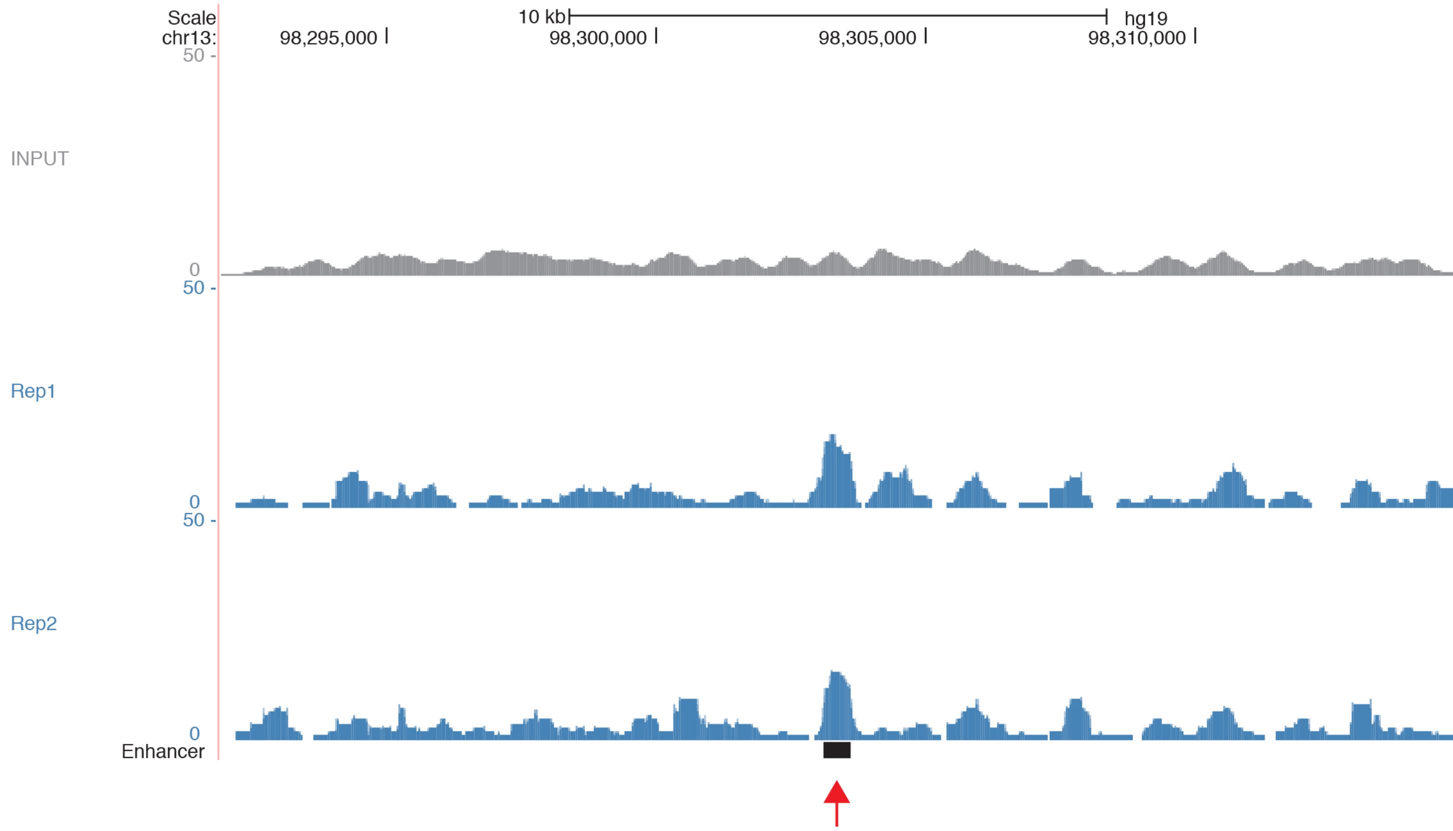

D

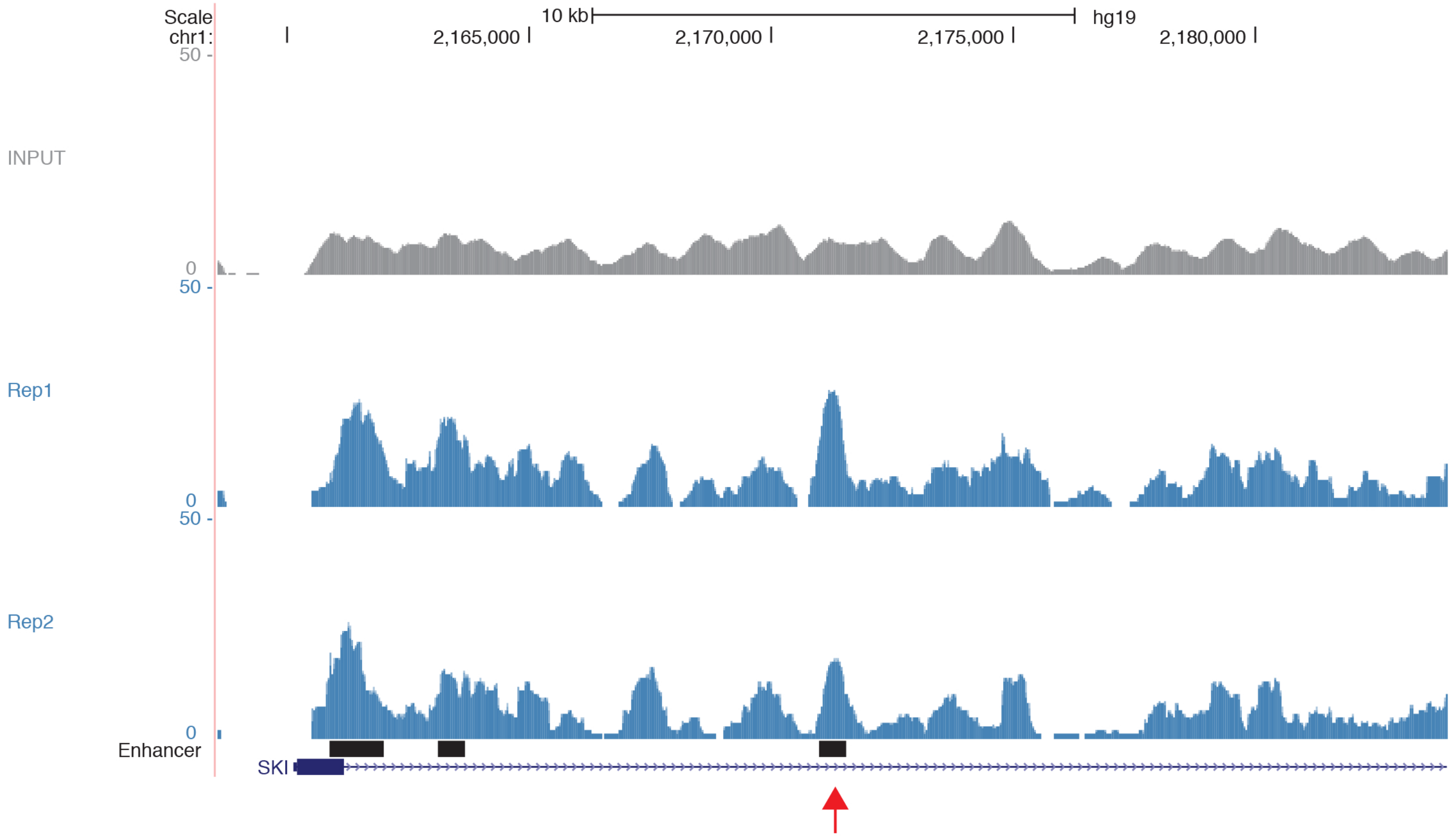

Figure S4: Snapshots of WHG-STARR-seq signal of strong and weak enhancers. Each blue track signifies normalized WHG-STARR-seq signal of each biological replicate. The gray track represents normalized WHG-STARR-seq signal of input library. Red arrows indicate the called enhancers. The activities of these enhancers are **A)** 6.85, **B)** 5.17, **C)** 4.00, and **D)** 3.10.

A

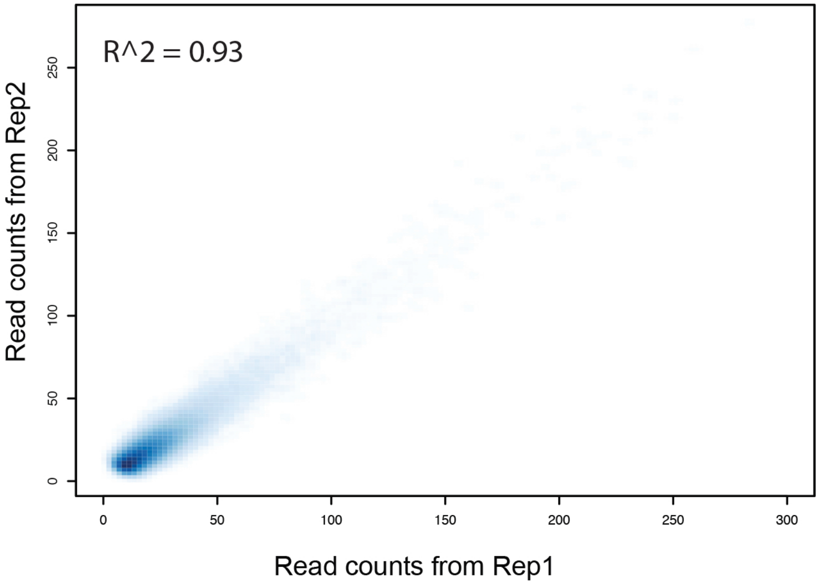

B

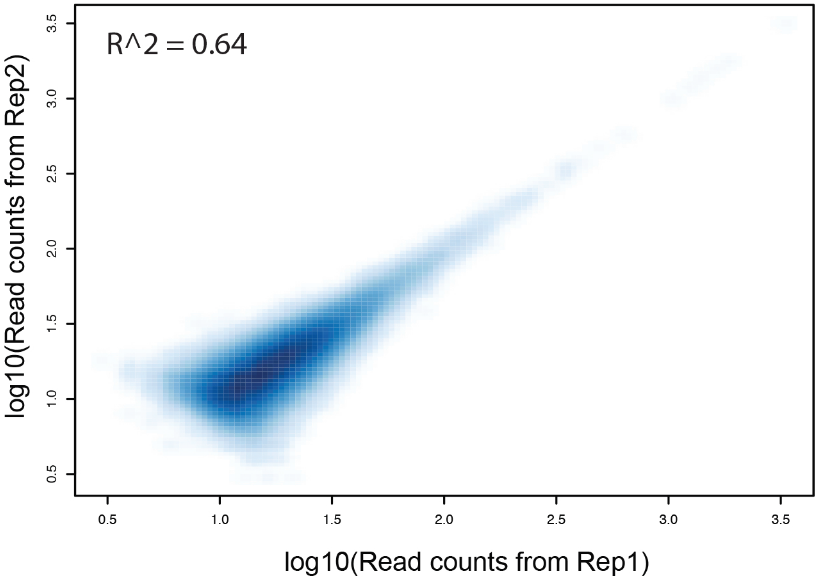

Figure S5: Reproducibility between biological replicates. Reproducibility plots between biological replicates were produced **A)** without taking the log of the read counts and **B)** with taking the log of the read counts. When comparing WHG-STARR-seq to other STARR-seq methods, the reproducibility of the assay appears comparable. What discrepancies do exist can be attributed to sequencing depth and amount of genomic space being investigated. Both STARR-seq (performed in Drosophila) and CapStarr-seq (capture-based screen performed in human cells) targeted far fewer genomic regions. As such, it's difficult to directly correlate and compare the assays, as WHG-STARR-seq targets the entire human genome (~23 times the size of the Drosophila genome and much higher than the 0.3% of the targeted investigative space interrogated with CapStarr-seq).

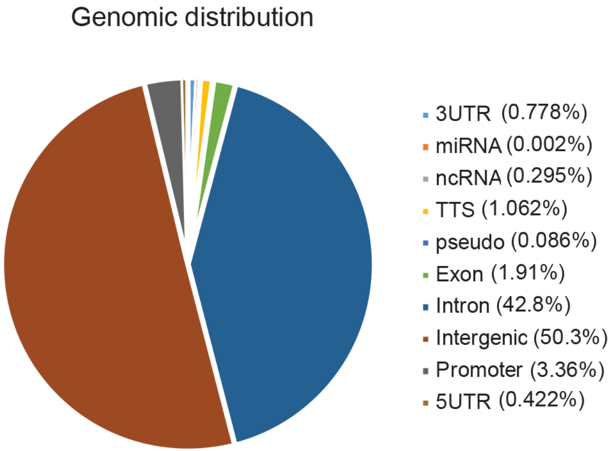

Figure S6: Genomic distribution of WHG-STARR-seq enhancers. Percentage values reflect the percent of total enhancers that fall within the respective genomic location. (3'UTR = 3' untranslated region; miRNA = micro RNA; ncRNA = non-coding RNA; TSS = transcriptional start site; pseudo = pseudogene; 5'UTR = 5' untranslated region; Promoter regions are defined as 1 kb upstream of TSS.)

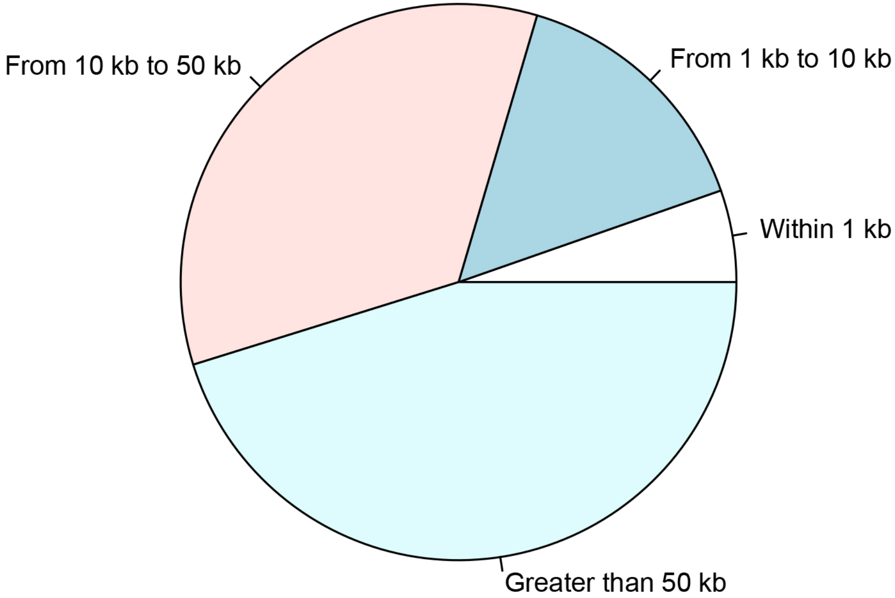

Figure S7: Relative distance of WHG-STARR-seq enhancers to nearby gene transcriptional start sites.

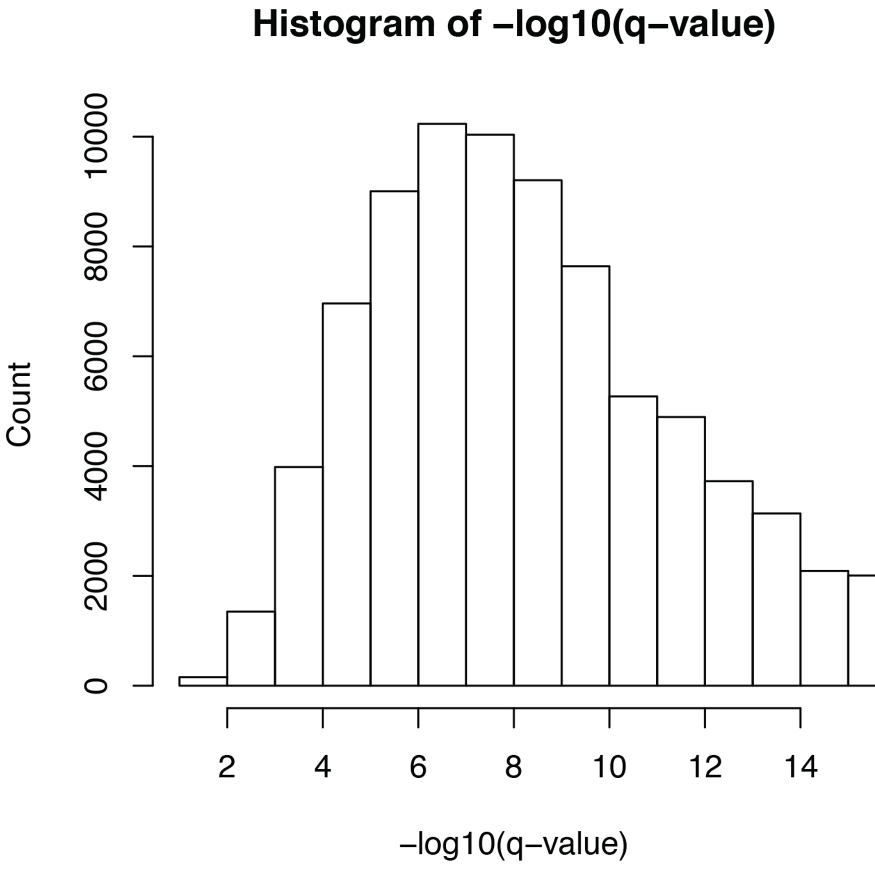

Figure S8: Distribution of MACS –log10(q-values) of called WHG-STARR-seq peaks

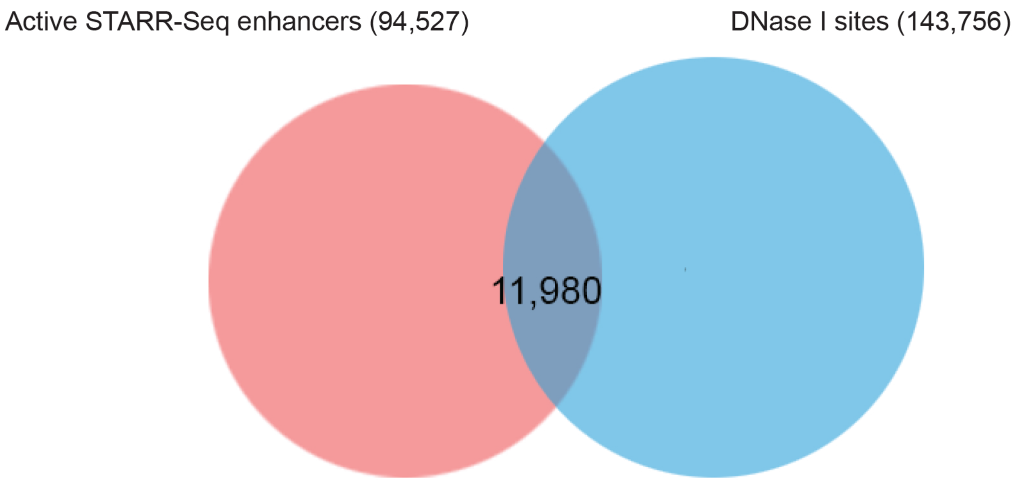

Figure S9: Venn diagram showing the overlap between WHG-STARR-seq enhancers and DNase I hypersensitivity sites

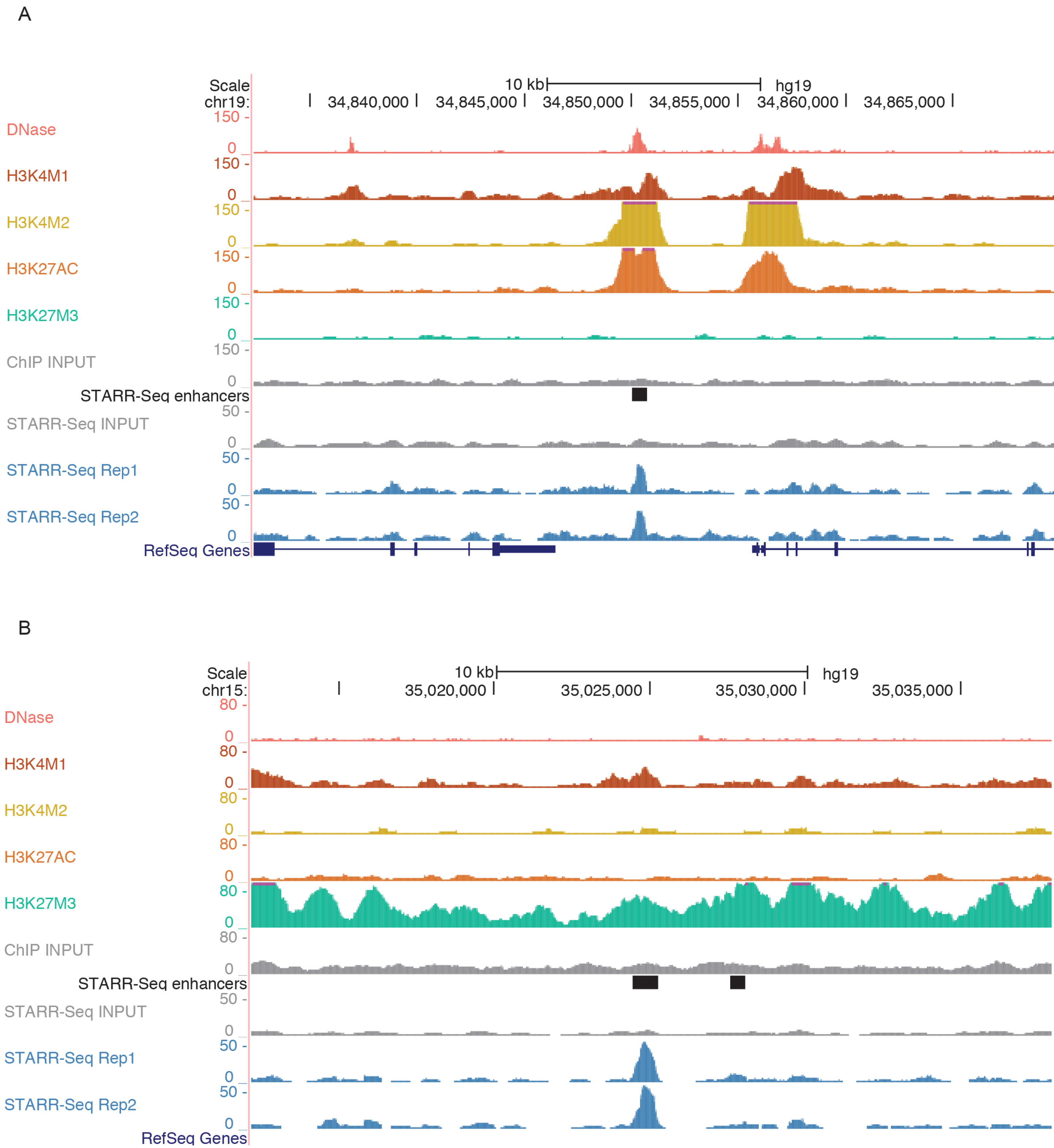

Figure S10: Snapshots of signal of various chromatin marks around WHG-STARR-seq enhancers. Enhancers located in **A)** Open chromatin regions and **B)** Closed chromatin regions.

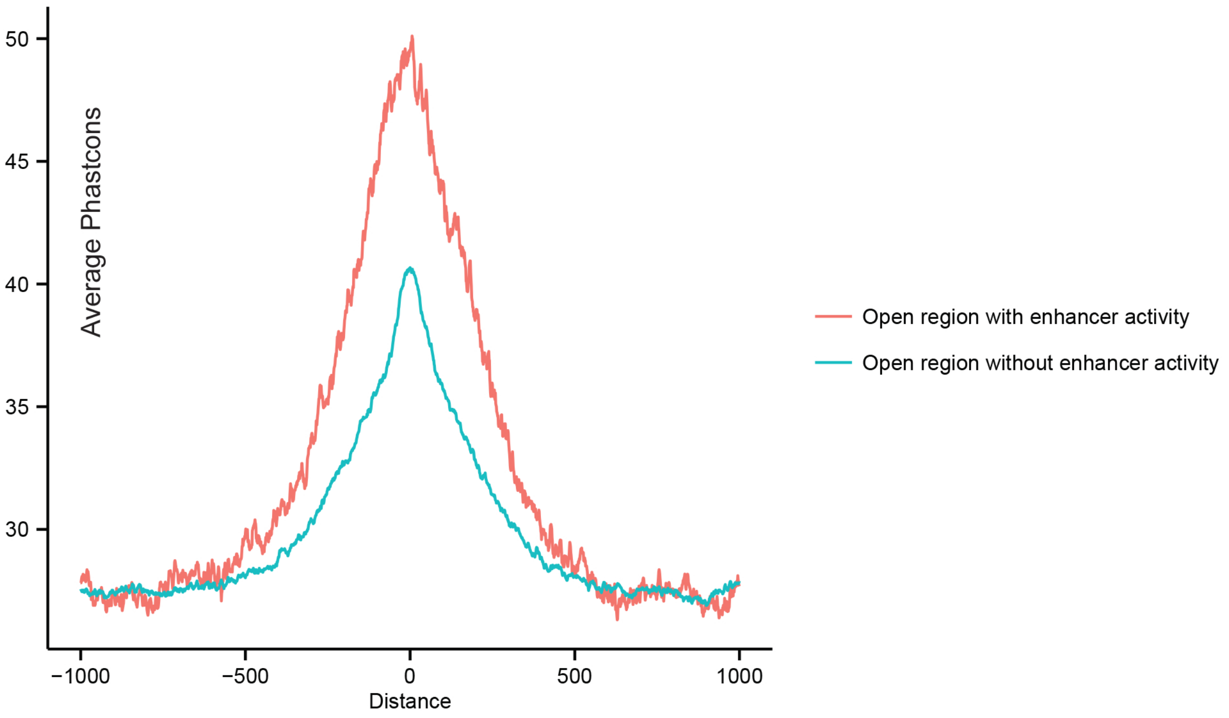

Figure S11: Conservation analysis on active/open WHG-STARR-seq enhancers and other non-active open chromatin regions.

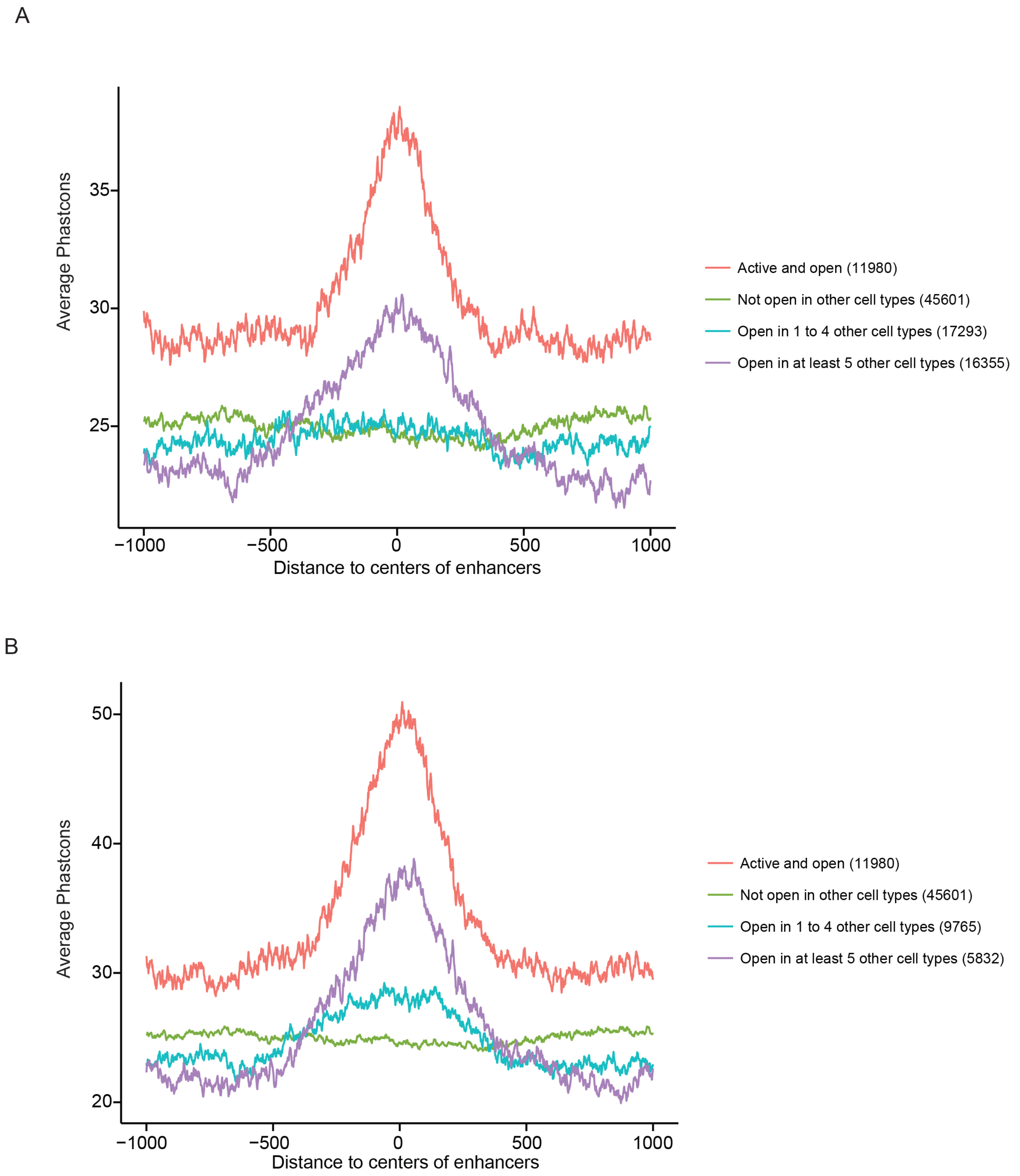

Figure S12: Conservation analysis on different categories of WHG-STARR-seq enhancers. A) Closed, active enhancer is defined as open in a cell type if it has at least 1-bp overlap with a DNase I site from that same cell type. **B)** Closed, active enhancers are defined as open in a cell type if it has at least 50% overlap with a DNase-I open region from that same cell type. The numbers in the parentheses represent the number of enhancers in each group. We are looking at the conservation profiles of +/- 1 kb regions around WHG-STARR-seq enhancers and the length of such enhancers is usually less than 1kb. Therefore, for this analysis, we only looked at active closed enhancers that do not have DHS sites within +/- 1kb away from their centers.
